# Comparative analysis of single- and dual-wavelength photodynamic therapy regimes with chlorin-based photosensitizers: animal study

**DOI:** 10.1117/1.JBO.25.6.063804

**Published:** 2019-12-23

**Authors:** Daria Kurakina, Aleksandr Khilov, Maria Shakhova, Natalia Orlinskaya, Ekaterina Sergeeva, Alina Meller, Ilya Turchin, Mikhail Kirillin

**Affiliations:** a Institute of Applied Physics RAS, Nizhny Novgorod, Russia; b Privolzhsky Research Medical University, Nizhny Novgorod, Russia

**Keywords:** photodynamic therapy, chlorin-based photosensitizers, fluorescence imaging, optical coherence tomography

## Abstract

Two pronounced absorption peaks in blue and red ranges of the chlorin-based photosensitizer (PS) absorption spectrum provide additional benefits in photodynamic therapy (PDT) performance. Differing optical properties of biological tissues in these ranges allow for both dual-wavelength diagnostics and PDT performance. We provide a comparative analysis of different PDT regimes performed with blue and red lights and their combination, with doses varying from 50 to $150\;{J/\mathrm{cm}}^{2}$. The study was performed on the intact skin of a rabbit ear inner surface, with the use of chlorin e6 as a PS. PDT procedure protocol included monitoring of the treated site with fluorescence imaging technique to evaluate PS accumulation and photobleaching, as well as with optical coherence tomography (OCT) to register morphological and functional responses of the tissue. Optical diagnostic observations were compared with the results of histopathology examination. We demonstrated that PDT procedures with the considered regimes induce weaker organism reaction manifested by edema in normal tissue as compared to irradiation-only exposures with the same light doses. The light doses delivered with red light induce weaker tissue reaction as compared to the same doses delivered with blue light only or with a combination of red and blue lights in equal parts. Results of *in-vivo* OCT monitoring of tissue reaction are in agreement with the results of histopathology study.

## 1 Introduction

Photodynamic therapy (PDT) is a modern minimally invasive treatment technique that has demonstrated efficiency for a wide range of clinical applications. PDT is based on the cytotoxic effect of singlet oxygen produced in biotissue as a result of irradiation with light of a specific spectral range of a photosensitizer (PS) delivered to the target area prior to the procedure.^1^^,^^2^ Being highly reactive with a short radius of action, the produced singlet oxygen causes damage in cells contacting with PS, thus providing a local impact with minimal effects on the surrounding normal tissue. Currently, PDT is actively employed for antitumor treatment^3^^,^^4^ and is being involved into anti-inflammatory treatment due to antimicrobial action, including cases of microbes resistant to traditional drug treatment.^5^ PDT also demonstrates positive aesthetic effects, such as skin rejuvenation, that have been well documented in a number of clinical trials.^6^ Both anti-inflammatory treatment and aesthetic medicine applications typically employ low-dose PDT regimes^7^ with total light doses not exceeding $150\;{J/\mathrm{cm}}^{2}$.

**Fig. 1:**
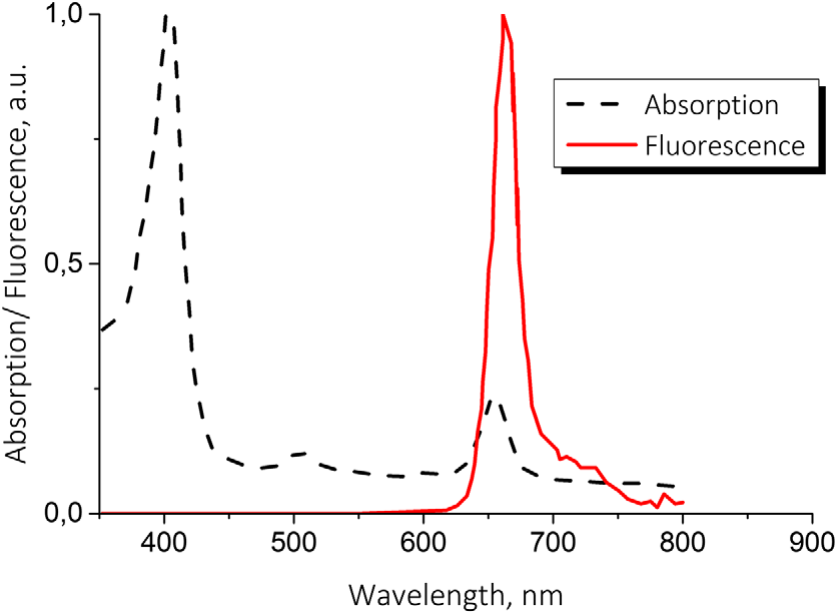
Chlorin e6 absorption and fluorescence spectra.

Chlorin-based PSs are now currently introduced into experimental medicine^8^^–^^10^ and clinical practice^11^^–^^14^ due to high efficiency and two pronounced absorption peaks in different bands of visible range (Fig. 1) that together with fluorescent properties provide additional opportunities for improving irradiation and monitoring protocols. Namely, the absorption spectrum of chlorin e6 features peaks at 402 and 662 nm that are attributed to blue and red bands. The majority of biotissues demonstrate significant dispersion in optical properties, especially in the absorption coefficient at these wavelengths.^15^ For example, for skin at 402 nm, the value of the absorption coefficient varies in the range of 0.3 to $1.3\;{\mathrm{mm}}^{-1}$, versus 0.02 to $0.3\;{\mathrm{mm}}^{-1}$ at 662 nm, while for the mucosa the absorption coefficient at 402 and 662 nm amounts to 0.48 and $0.01\;{\mathrm{mm}}^{-1}$, respectively.^16^^,^^17^ Such a difference results in different distribution of the absorbed light dose within the tissue, since blue light is absorbed primarily in the superficial layers, while red light penetrates deeper.^18^^,^^19^ This provides an additional opportunity to control the impact depth in the course of a PDT procedure.

Protocols for antitumor PDT frequently imply intravenous PS injection^3^^,^^4^ and its selective accumulation in tumor due to increased microcirculatory activity. In case of deeper tumor locations, obviously, red-light irradiation is preferable. However, particular oncological applications, together with antimicrobial and antiaging PDT applications, involve topical PS administration.^3^^,^^20^^,^^21^ This option is especially preferable when superficial action is required and employment of blue-light irradiation may be efficient.

Irradiation with blue light may provide additional antimicrobial effect,^22^^,^^23^ which is important in PDT treatment of acute and chronic inflammatory diseases. Moreover, blue light was demonstrated to stimulate neoangiogenesis at low doses, which is important for regenerative medicine.^24^ In particular cases, drug treatment strategy against inflammatory diseases strongly depends on the proper selection of a drug that is efficient against a particular causative agent. When the causative agent is not determined, the drug treatment may show weak efficacy, while PDT acts as a wide-range aid and can serve a method of choice in this situation.

Most of PDT protocols with chlorin-based PSs, especially, tumor treatment protocols, employ irradiation wavelengths around 662 nm to ensure deeper light penetration.^8^^–^^12^ There are several reports on the application of blue spectral range with such PSs as aminolevulinic acid or methyl aminolevulinate,^6^^,^^20^^,^^25^^,^^26^ which also have several absorption peaks including red and blue bands; however, the reported applications with chlorin-based PS are very limited.^14^^,^^24^

In our previous paper,^24^ we reported on a clinical study of antimicrobial PDT of pharynx chronic inflammatory diseases with topical application of chlorin-based PS and irradiation wavelength of 405 nm. The employed PDT protocol revealed no side effects or complications, while microbiological study after PDT revealed no pathogenic bacteria. However, the lack of information on the features of PDT performance with chlorin-based PS at 405 nm causes the necessity of further investigation.

Two specific absorption peaks in combination with fluorescent properties of chlorin e6 PS provide additional diagnostic opportunities for fluorescence imaging (FI). FI is widely employed for monitoring the PS accumulation and distribution in biotissue prior to a PDT procedure^1^^,^^27^^–^^29^ and for evaluation of PS photobleaching in the course of the procedure.^30^^,^^31^ Fluorescence excitation at two different wavelengths from the red and blue spectral ranges provides the opportunity to detect fluorescence response of PS from different depths depending on the penetration of the probing radiation into the tissue. The ratio of the fluorescence response values at these wavelengths yields the information about the penetration depth of PS upon topical administration, since prevailing response at blue-light excitation indicates accumulation in superficial layers, while prevailing response at red-light excitation indicates deeper PS penetration.^32^^,^^33^

Optical coherence tomography (OCT) with angiographic modality provides complementary diagnostic opportunities in noninvasive immediate monitoring of PDT response such as visualization of structural and vascular changes caused by a PDT procedure. For instance, optical coherence angiography (OCA) was used to monitor treatment response following vascular-targeted PDT,^34^^,^^35^ and a criterion of PDT success based on OCA images of tumor and peritumor areas acquired 24 h post treatment was suggested.

The aim of this study is to compare the effects of different PDT regimes after topical administration of chlorin-based PS and the irradiation-only exposures at the wavelengths of 405 and 660 nm on intact tissue based on both results of noninvasive optical monitoring and histopathology inspection. Considered doses are in the range of 50 to $150\;{J/\mathrm{cm}}^{2}$ for blue- and red-light irradiations separately and combined doses of 100 and $150\;{J/\mathrm{cm}}^{2}$ with equal contribution of red and blue irradiations (50+50 and $75+75\;{J/\mathrm{cm}}^{2}$, respectively). The dual-wavelength PDT regimes are rarely reported, although they have the potential due to the impact to different depths in tissue.^14^

## 2 Materials and Methods

### 2.1 Animals

A total of 12 female Russian Chinchilla rabbits were enrolled into the study. The inner surface of the rabbit ear was chosen as a treated area due to accessibility and convenient imaging performance. For each rabbit, three areas in the inner surface of each ear were selected either for PDT treatment or for irradiation-only exposure. Prior to the PDT procedure, the rabbits were narcotized with Zoletil/XylaVet in the amount of 0.2  ml/kg injected intravenously. The animal studies were approved by the Ethics Committee of Privolzhsky Research Medical University (Protocol #7, 03.07.2017).

### 2.2 Photodynamic Therapy Procedures

The doses of 50, 75, 100, and $150\;{J/\mathrm{cm}}^{2}$ were considered for red- and blue-light irradiations separately (these regimes are further referred to in the text as PDT_r50, PDT_r75, PDT_r100, and PDT_r150 for red light regimes, and PDT_b50, PDT_b75, PDT_b100, and PDT_b150 for blue light regimes, respectively), as well as the combined doses of 100 and $150\;{J/\mathrm{cm}}^{2}$ with equal contribution (50+50 and $75+75\;{J/\mathrm{cm}}^{2}$, respectively) of red and blue irradiations (referenced as PDT_rb100 and PDT_rb150, respectively). The same doses were delivered to the animals from the irradiation-only group without PS administration (these regimes are further referred to with abbreviation IRR instead of PDT). The nomenclature of the considered regimes is summarized in Table 1.

**Table 1:** Nomenclature of the considered exposure regimes.

Delivered dose ($J/{\mathrm{cm}}^{2}$)	Red light (λ=660 nm)		Blue light (λ=405 nm)		Combination with equal doses (λ=660+405 nm)	
	PDT procedure	Irradiation-only	PDT procedure	Irradiation-only	PDT procedure	Irradiation-only
50	PDT_r50	IRR_r50	PDT_b50	IRR_b50	—	—
75	PDT_r75	IRR_r75	PDT_b75	IRR_b75	—	—
100	PDT_r100	IRR_r100	PDT_b100	IRR_b100	PDT_rb100 (PDT_r50 + PDT_b50)	IRR_rb100 (IRR_r50 + IRR_b50)
150	PDT_r150	IRR_r150	PDT_b150	IRR_b150	PDT_rb150 (PDT_r75 + PDT_b75)	IRR_rb150 (IRR_r75 + IRR_b75)

Each particular regime was applied to a separate rabbit ear in the three selected areas. The chlorin e6 PS “Revixan derma” (Revixan Ltd., Russia) was applied topically to the treated area in the amount of 0.1 ml and was distributed evenly with a cotton swab over $2\times 3\;{\mathrm{cm}}^{2}$ of tissue surface. In 30 min after application, the rest of the PS was removed from the tissue surface, also with a cotton swab. Prior to the PDT procedure, all the surrounding tissues except the treated area were covered by a reflecting tape to avoid their direct irradiation. The irradiation was performed with the PDT device “Harmonia” (Laser MedCenter Ltd, Russia) equipped with light-emitting diode (LED) arrays with the wavelengths of 405 and 660 nm, and the fluence rate at the tissue surface was $200\;{\mathrm{mW}/\mathrm{cm}}^{2}$ for both wavelengths.

### 2.3 Optical Diagnostics Modalities

Two optical imaging modalities were employed to monitor the PDT procedure and the tissue reaction after the procedure: OCT and FI. The OCT-1300E device (IAP RAS, BioMedTech Ltd., Russia), operating at the central wavelength of 1300 nm and capable of providing three-dimensional OCT imaging with axial spatial resolution of 15  μm combined with OCT angiography, was employed to monitor the structural and functional changes after the exposure. OCT angiography concept implemented in the device is based on the analysis of decorrelation of OCT image speckle pattern induced by blood flow within tissue.^36^^,^^37^ The device is equipped with the contact probe, and the probe pressure to the tissue was controlled by a custom-designed holder to avoid effects of tissue compression on OCT images.^38^ OCT imaging of the target area was performed prior to and immediately after the exposure and in 1, 4, and 7 days after the procedure (in 24, 96, and 168 hours, respectively).

Monitoring of PS accumulation in the target area and its photobleaching in the course of the PDT procedure was performed by an FI device (IAP RAS, Russia) with probing wavelengths of 405 and 660 nm corresponding to the absorption bands of chlorin e6, and fluorescence detection in the range of 702 to 842 nm.^33^^,^^39^ The device provides two-dimensional fluorescence images of the target area at both excitation wavelengths. This approach allows to indirectly estimate the PS penetration depth and the range of PDT action depths basing on the red-to-blue signal ratio dynamics $R_{\lambda }=I_{f660}/I_{f405}$, where $I_{f660}$ and $I_{f405}$ are the fluorescence intensities averaged over the region of interest upon excitation at wavelengths of 660 and 405 nm, respectively.^32^^,^^33^ FI was performed on the day of the PDT procedure only. The drop in the fluorescence signal, indicating PS photobleaching, was considered as an indicator of the procedure efficiency. For quantitative characterization, the photobleaching efficiency was calculated as $\mathrm{PE}=(I_{f1}-I_{f2})/I_{f1}\cdot 100\%$, where $I_{f1}$ and $I_{f2}$ are the fluorescence intensities averaged over the region of interest before and after the PDT procedure, respectively.

Monitoring of the tissue temperature prior to and immediately after the PDT procedure was performed by infrared (IR) thermometer (Optris, Germany).

### 2.4 Histopathological Studies

Histopathological studies were performed along with the optical monitoring, in order to verify and complement the noninvasive diagnostics data. Biopsy samples were taken from the target sites in 1, 4, and 7 days after the PDT procedure following OCT examination and were further subjects for hematoxylin and eosin (H&E) staining. Morphological studies were performed employing Leica DM 2500 microscope. The following morphological parameters were primarily analyzed: signs of edema and inflammation and the area of the newly formed vessel.

### 2.5 Monte Carlo Simulations of Absorbed Light Dose

Monte Carlo technique is a common tool for evaluation of light dose distribution in PDT.^40^ Monte Carlo technique is based on the modeling of a large number of random photon trajectories in turbid media followed by the statistical analysis of the collected data. A custom-developed MATLAB-based implementation^24^^,^^33^ of Monte Carlo algorithm was employed to simulate the distribution of absorbed light dose in a rabbit ear for irradiation at 405 and 660 nm. Simulations were performed for a planar three-layered model of a rabbit ear with the thickness of 1 mm, where top and bottom layers correspond to the skin tissues and the intermediate layer corresponds to the cartilage. Owing to the lack of optical properties data for rabbit skin and cartilage, optical properties of human skin and cartilage^16^^,^^41^ were employed for the preliminary assessment of light distribution depending on the irradiation wavelength. The values of absorption and reduced scattering coefficients employed for simulations are summarized in Table 2.

**Table 2:** The optical properties of skin and cartilage (absorption $\mu_{a}$ and reduced scattering $\mu_{s}^{\prime }$ coefficients) used for Monte Carlo simulations.^16^^,^^41^

	Thickness, mm	n	$\mu_{a}$, ${\mathrm{mm}}^{-1}$		$\mu_{s}^{\prime }$, ${\mathrm{mm}}^{-1}$	
			405 nm	660 nm	405 nm	660 nm
Skin	0.4	1.45	1.3	0.3	5.4	4.6
Cartilage	0.2	1.4	0.08	0.01	3	1

## 3 Results and Discussion

### 3.1 Fluorescence Monitoring of the Photodynamic Therapy Procedure

**Fig. 2:**
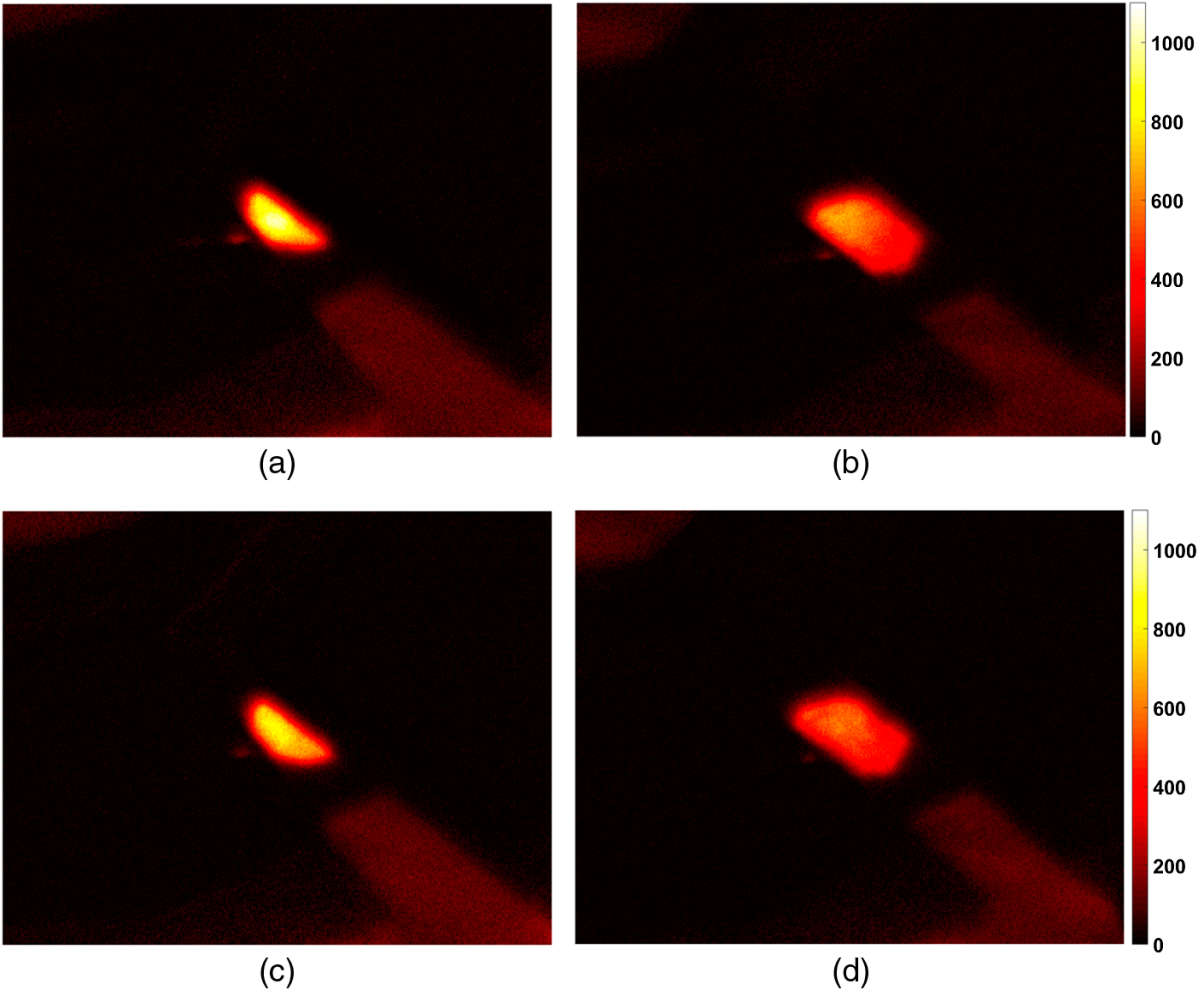
Typical fluorescence images of the treated area captured before [(a), (c)] and after [(b), (d)] the PDT_b50 procedure ($50\;{J/\mathrm{cm}}^{2}$, 405 nm). Fluorescence is collected after excitation at the wavelengths of 405 nm [(a), (b)] and 660 nm [(c), (d)].

**Fig. 3:**
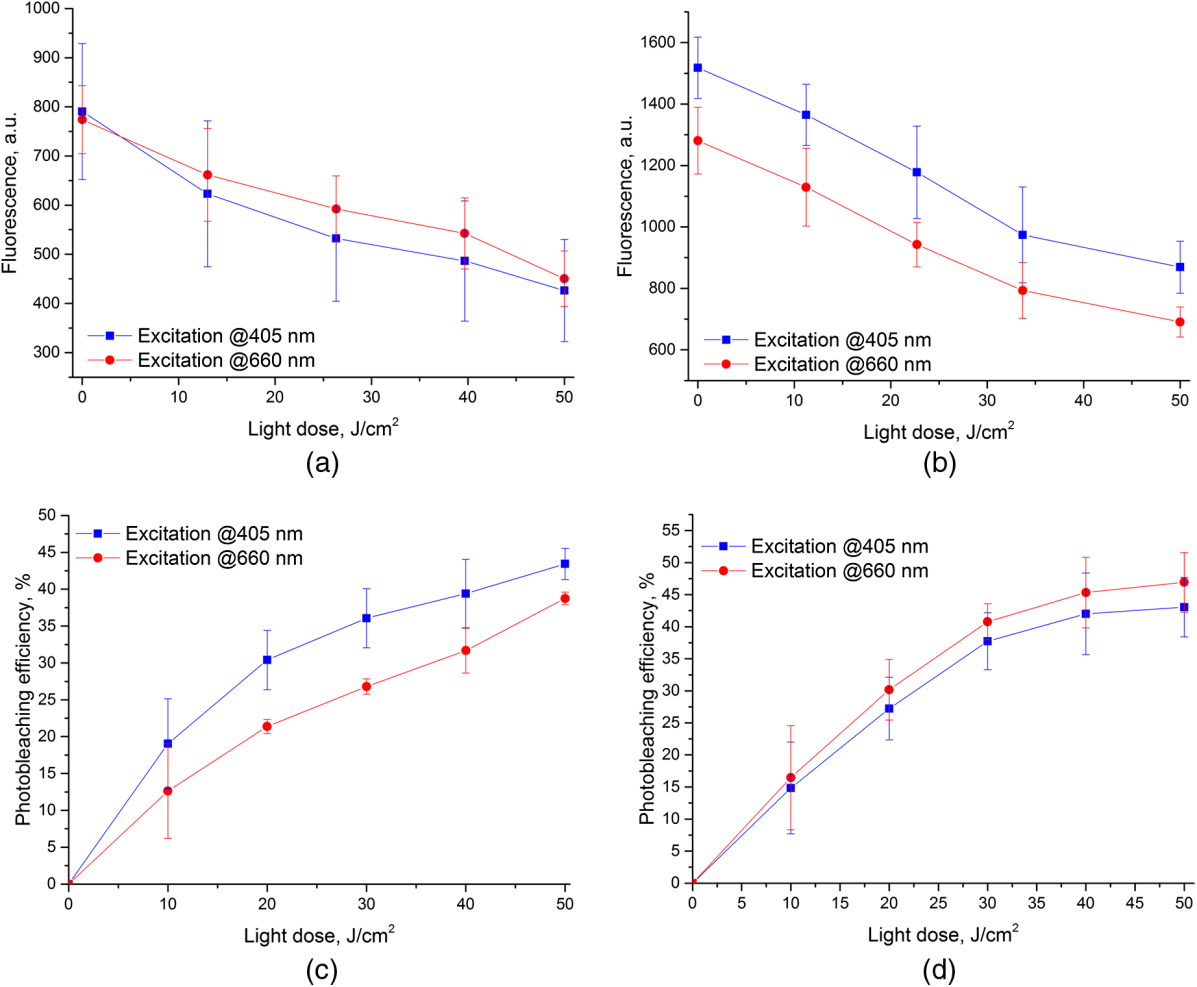
Averaged dynamics of fluorescence signals at two excitation wavelengths during (a) PDT_b50 ($50\;{J/\mathrm{cm}}^{2}$, 405 nm) and (b) PDT_r50 ($50\;{J/\mathrm{cm}}^{2}$, 660 nm) procedures and averaged dependences of PS photobleaching efficiency on delivered light dose during (c) PDT_b and (d) PDT_r procedures (n=3).

The primary aim of the FI in a PDT procedure assistance is monitoring of PS accumulation in tissue and its photobleaching in the course of light irradiation. Figure 2 shows typical fluorescence images of treated area captured before and after PDT_b50 procedure upon excitation at two wavelengths of 405 and 660 nm and fluorescence registration in the range of 702 to 842 nm. Dual-wavelength fluorescence monitoring provides information from different measurement volumes and allows estimating the dynamics of the PS in-depth distribution. Figure 3 demonstrates the averaged evolution of a fluorescence response from the treatment site in the course of the PDT procedure. The fluorescence signal for each measurement is averaged over the entire fluorescing area in the registered image. The results are presented for PDT_b50 [Fig. 3(a)] and PDT_r50 [Fig. 3(b)] regimes. For each procedure, both fluorescence responses at red (λ=660  nm) and blue (λ=405  nm) excitation wavelengths are given.

Dynamics of the fluorescence signal at both excitation wavelengths demonstrate monotonous decrease with the delivered light dose, indicating PS photobleaching in the course of irradiation. For all the considered cases, the delivered light dose of $50\;{J/\mathrm{cm}}^{2}$ resulted in the photobleaching efficiency of PE=41.9%±6.1% and PE=43.2%±7.2% for the fluorescence excitation at the wavelengths of 405 nm and 660 nm, respectively. The achieved PE values confirm the efficiency of the performed procedure, since the level of 40% is typically considered as a success.^28^ Averaged photobleaching efficiencies as the function of the delivered light dose at the wavelength of 405 and 660 nm are shown in Figs. 3(c) and 3(d), respectively. One can see that the photobleaching efficiency of 40% is achieved on the average after the dose of $50\;{J/\mathrm{cm}}^{2}$. The corresponding photobleaching rates α expressed in inverse dose units ${\left(J/{\mathrm{cm}}^{2}\right)}^{-1}$ and derived from the approximation of the fluorescence decay with the delivered dose *D* by the expression $I_{f}(D)=I_{f1}\exp(-\alpha D)$ are summarized in Table 3.

**Table 3:** Rates α of PDT-induced photobleaching of fluorescence excited at the two wavelengths.

PDT regime	α for excitation at 405 nm, ${\left(J/{\mathrm{cm}}^{2}\right)}^{-1}$	α for excitation at 660 nm, ${\left(J/{\mathrm{cm}}^{2}\right)}^{-1}$
PDT_b (405 nm)	0.011	0.009
PDT_r (660 nm)	0.014	0.017

**Fig. 4:**
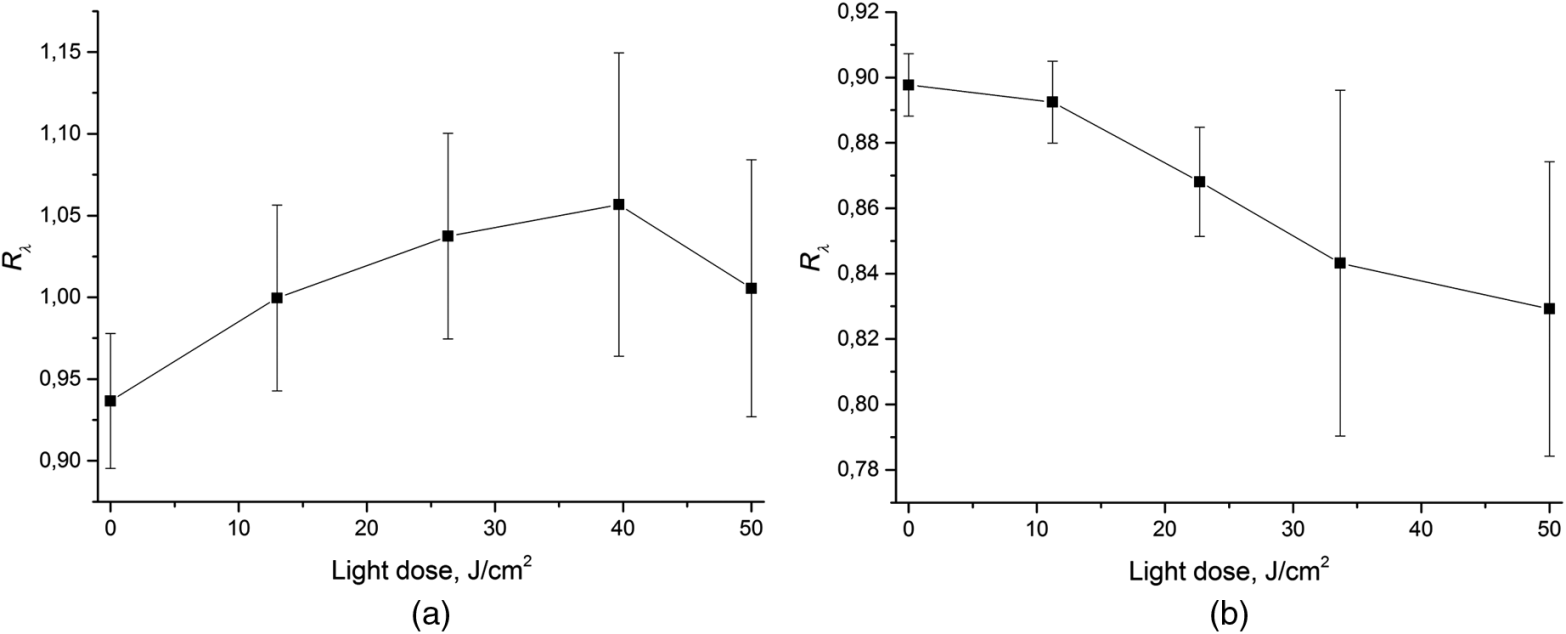
Averaged evolution of red-to-blue signal ratio $R_{\lambda }$ during (a) PDT_b50 ($50\;{J/\mathrm{cm}}^{2}$, 405 nm) and (b) PDT_r50 ($50\;{J/\mathrm{cm}}^{2}$, 660 nm) procedures (n=3).

Figure 4 shows the averaged evolution of red-to-blue signal ratio $R_{\lambda }$ during PDT_b50 and PDT_r50 procedures shown in Figs. 4(a) and 4(b). PDT with blue light [Fig. 4(a)] results in the initial increase of $R_{\lambda }$ associated with faster photobleaching in the superficial layers due to smaller penetration depth of blue light as compared to red one. On the contrary, PDT procedure with red light [Fig. 4(b)] is accompanied by a monotonous decrease of $R_{\lambda }$ indicating faster photobleaching in deeper tissue layers. Note that the large standard deviations are determined by the differences in the absolute values, while the same trends are observed in all individual cases. In accordance with Monte Carlo simulations performed for the considered three-layered model using the previously developed methodology,^33^ the values of $R_{\lambda }$ of ∼0.8 to 1.1 correspond to the typical penetration depth of 0.4 to 0.8 mm, which is in agreement with typical impact depths in a rabbit ear.

Thus, dual wavelength fluorescence monitoring allowed to trace the PS penetration depth and the range of PDT action depths by the dynamics of the PS photobleaching in the course of irradiation with red and blue lights.

### 3.2 Temperature Monitoring in Course of a Photodynamic Therapy Procedure

**Fig. 5:**
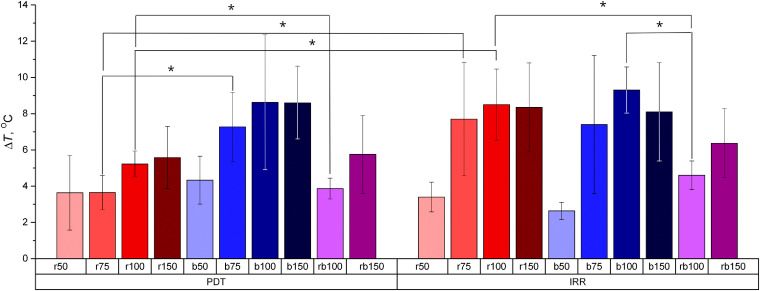
Temperature increase ΔT as a result of different exposure regimes (Table 1). Asterisk (*) indicates statistically significant difference (p<0.05).

Temperature is an essential parameter of the treatment site to be monitored in course of a PDT procedure, since a substantial temperature increase may lead to an additional undesirable effect on the tissue. It is of a special importance when performing a PDT procedure with the blue light, since light absorption in tissue in this case is much larger as compared to the red light. The temperature of the rabbit ear prior to exposure amounted to 30.5±2.1°C, and the temperature after the exposure did not exceed 39.6°C for all the considered regimes. Figure 5 summarizes the temperature increase ΔT as a result of exposure for all the considered regimes. The values are averaged over all considered treatment sites.

One can see that all the exposure regimes induce statistically significant temperature increase. The temperature increase for the PDT regimes employing blue light is higher as compared to those with red light. However, for IRR regimes, such trend was not observed. Surprisingly, the combination regimes PDT_rb100 and IRR_rb100 provide smaller temperature increase as compared to both red and blue mono-wavelength exposures for both PDT and IRR regimes with the equivalent dose. This effect can be associated with additional temperature decrease during switching the irradiation sources from one wavelength to another. Statistically significant differences between corresponding exposure regimes with equivalent doses are marked with asterisks.

### 3.3 Morphological Features of Rabbit Ear

**Fig. 6:**
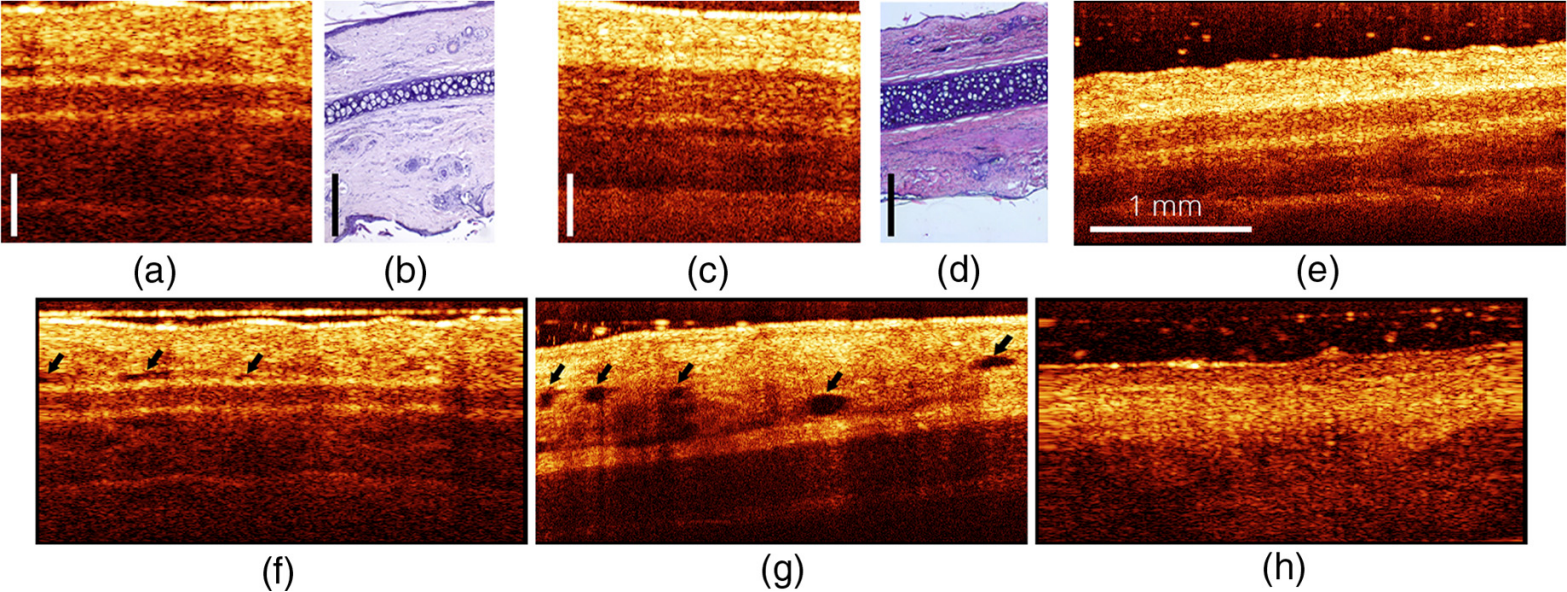
OCT [(a), (c)] and histology [(b), (d)] images of thin [(a), (b)] and thick [(c), (d)] compartments of a rabbit ear. (e)–(h) Typical OCT images of rabbit ear (e) in norm and with (f) manifestations of edema, (g) activation of lymphatic system, and (h) severe changes. Scale bars in (a)–(d) are 200  μm. Black arrows in (f) and (g) represent corresponding structural changes.

To study both tissue morphological and functional responses to a PDT procedure, OCT monitoring of the treated site was performed prior to the PS application, immediately after PDT procedure, and in 1, 4, and 7 days after the procedure. Two-dimensional OCT images (B-scans) demonstrate typical rabbit ear structure represented by central cartilage layer covered by skin layers on the dorsal and ventral surfaces [Figs. 6(a) and 6(c)].

Figure 6 shows the comparison of the structure of the thin and thick compartments of a rabbit ear obtained by OCT [Figs. 6(a) and 6(c)] and histological examination [Figs. 6(b) and 6(d)]. The thin compartment of a rabbit ear has a total thickness of about 0.5 to 0.8 mm and can be visualized by OCT over the entire depth [Fig. 6(a)]. The thickness of the cartilage layer is about 80 to 100  μm. In the region of thick compartment of a rabbit ear with a total thickness of about 0.6 to 1.5 mm the cartilage thickness is significantly higher (about 160 to 230  μm) [Fig. 6(c)], which in some cases may affect the visualization of the skin layer under the cartilage [for example, see Fig. 6(g)].

OCT imaging allows monitoring tissue reaction to the PDT procedure typically manifested by edema, activation of lymphatic system, or severe changes manifested by alterations in the observed layered structure. Figure 6(e) demonstrates an OCT image of an unaltered rabbit ear. Edema is usually manifested in OCT images by dark areas elongated parallel to the tissue surface and associated with the income of extracellular water to the region under exposure [Fig. 6(f)], while activation of lymphatic system appears as highly contrasted dark oval-shaped inclusions originating from lymph vessels filled with transparent lymph [Fig. 6(g)]. Severe changes are manifested by disappearance of the layered structure associated with the morphological alterations and the appearance of a rigid scab [Fig. 6(h)].

### 3.4 Numerical Simulations of Absorbed Dose Distribution

**Fig. 7:**
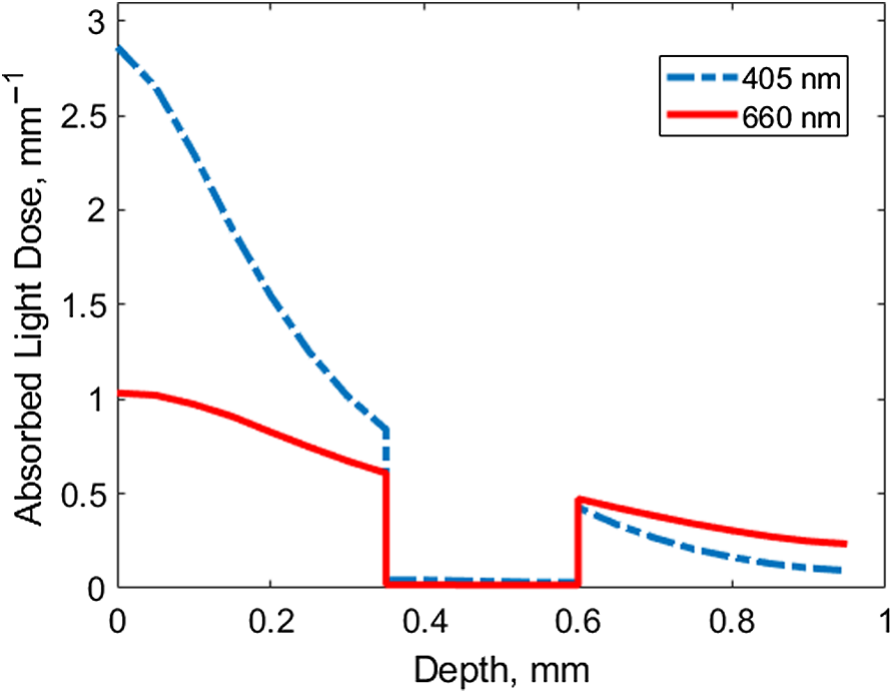
Simulated in-depth distribution of the absorbed light dose in a rabbit ear for wavelengths of λ=405  nm and λ=660  nm represented as the ratio of the photon number absorbed per unit volume to the photon number launched per unit area (in ${\mathrm{mm}}^{-1}$).

To estimate the effect of the irradiation wavelength on the light distribution, the in-depth profiles of the absorbed light dose at the wavelengths of 405 and 660 nm were numerically simulated (Fig. 7) for the planar three-layered model of a rabbit ear corresponding to the typical structural OCT images. At the wavelength of λ=405  nm, a great part of the light energy is absorbed in the superficial layer, while for λ=660  nm, the in-depth decay of the absorbed light dose with depth is smoother. The value of the absorbed light dose in the upper skin layer is higher for λ=405  nm. Both these effects are explained by the significant dispersion of the absorption coefficient values with the wavelength.

### 3.5 Comparative Analysis of Photodynamic Therapy Regimes

**Fig. 8:**
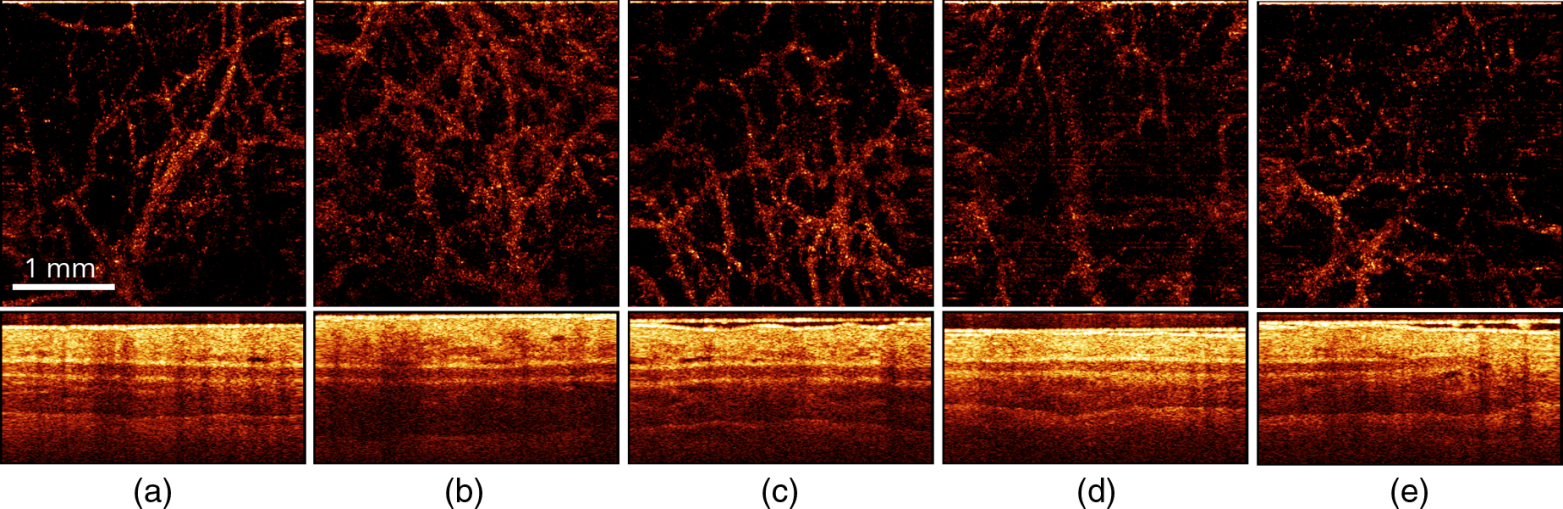
(Top) Angiographic *en-face* projection and (bottom) structural OCT images of rabbit ear (thin compartment) (a) prior to, (b) immediately after, (c) in 1, (d) 4, and (e) 7 days after the PDT_r50 procedure ($50\;J/{\mathrm{cm}}^{2}$, 660 nm). The size of top and bottom images is 3×3 and 3×1  mm, respectively.

Typical results of OCT monitoring of the PDT_r50 procedure outcome performed within the thin compartment of a rabbit ear are shown in Fig. 8, for both angiography and structural modalities. Such regime is typical for the antimicrobial/antiaging PDT procedure.^7^ One can see that OCT monitoring does not reveal any pronounced reaction of the tissue to the procedure, except weak manifestation of edema immediately after and in 1 day after the procedure. OCT angiography provides clear visualization of the vessel net for every observation period, indicating no disturbance in tissue microcirculation. Similar observations were obtained for IRR_r50 exposure, as well as for both PDT_b50 and IRR_b50 exposures.

**Fig. 9:**
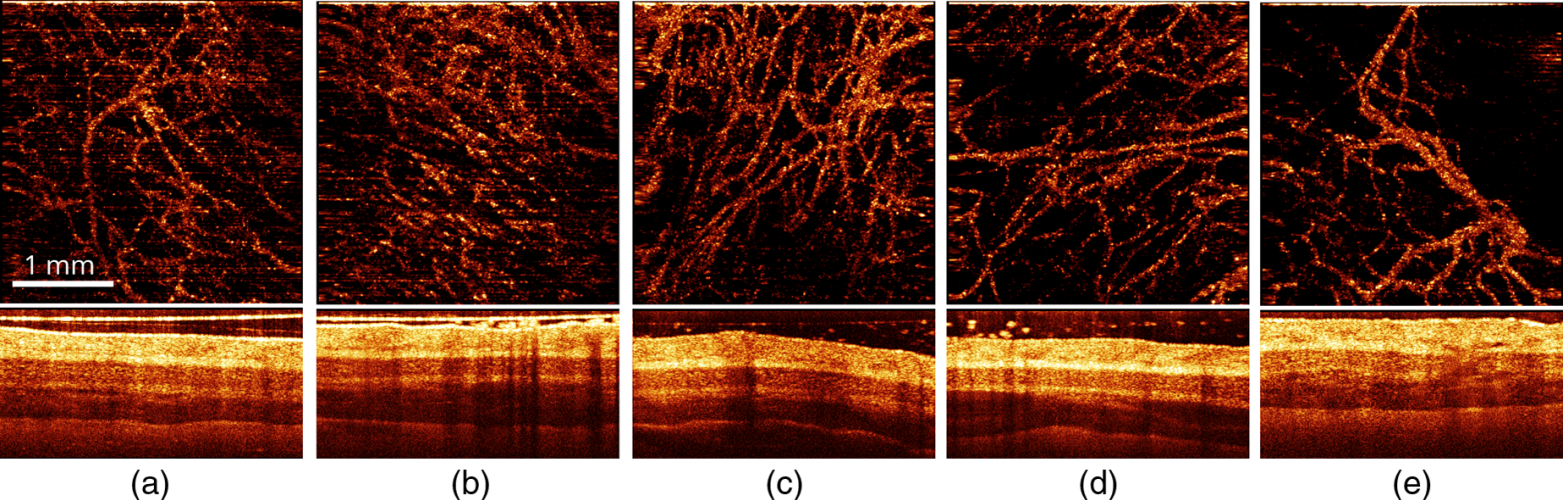
(Top) Angiographic *en-face* projection and (bottom) structural OCT images of a rabbit ear (thick compartment) (a)prior to, (b) immediately after, in (c) 1,(d) 4, and (e) 7 days after IRR_b75 exposure (irradiation-only, $75\;{J/\mathrm{cm}}^{2}$, 405 nm). The size of top and bottom images is 3×3 and 3×1  mm, respectively.

Increase of the delivered dose to $75\;J/{\mathrm{cm}}^{2}$ did not reveal any pronounced differences in the manifestation of tissue reaction to PDT and IRR regimes at both wavelengths. Figure 9 demonstrates OCT monitoring of IRR_b75 exposure applied to the thick compartment of a rabbit ear.

**Fig. 10:**
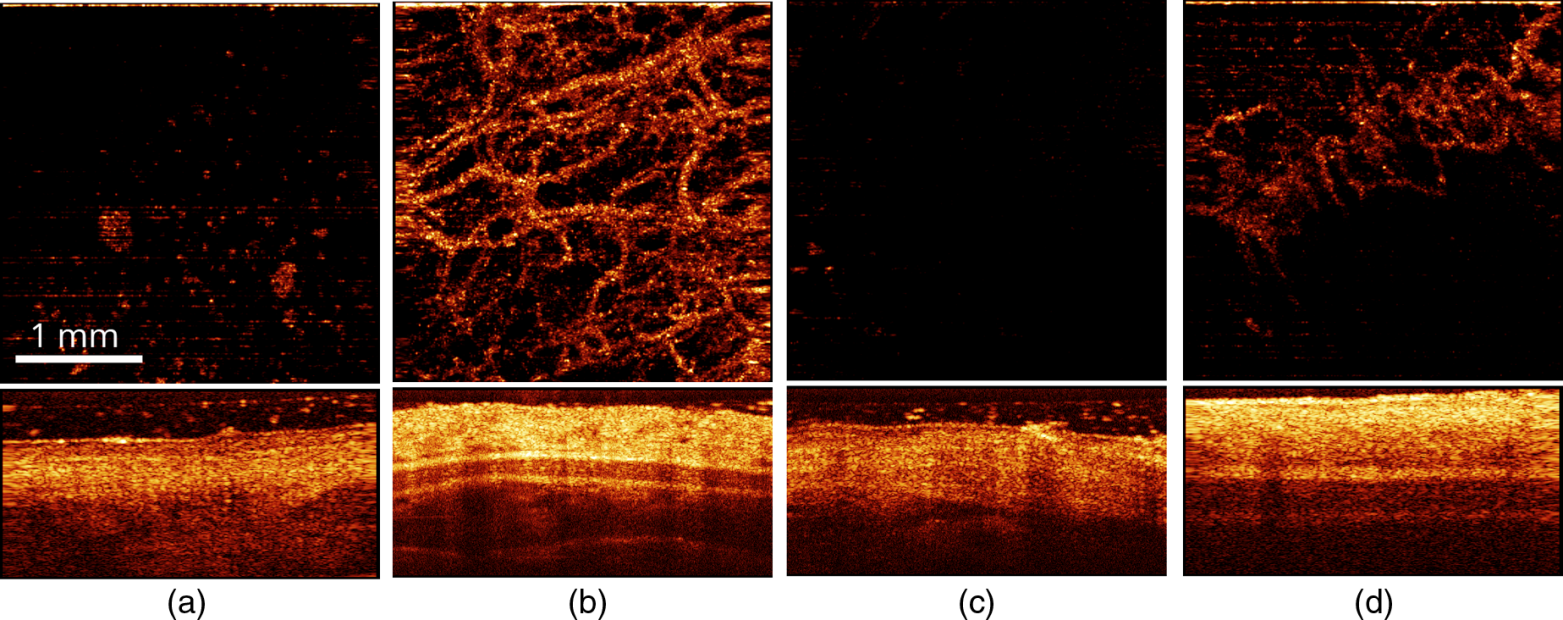
(Top) Angiographic *en-face* projection and (bottom) structural OCT images of rabbit ear obtained from different animals in (a) 4 and (b) 7 days after the PDT_b100 procedure ($100\;{J/\mathrm{cm}}^{2}$, 405 nm) and in (c) 4 and (d) 7 days after PDT_b150 procedure ($150\;{J/\mathrm{cm}}^{2}$, 405 nm). The size of top and bottom images is 3×3 and 3×1  mm, respectively.

Further increase of the delivered dose up to 100 and $150\;{J/\mathrm{cm}}^{2}$ demonstrated different reactions to the same procedure in different animals. For the PDT_b100 and PDT_b150 procedures, some animals demonstrated severe changes in structural OCT images and stasis in angiographic images in 4 days after the procedure [Figs. 10(a) and 10(c)], while other animals demonstrated no significant alterations and preservation of microcirculation in 4 days (not shown) and in 7 days [Figs. 10(b) and 10(d)] after the procedure.

**Fig. 11:**
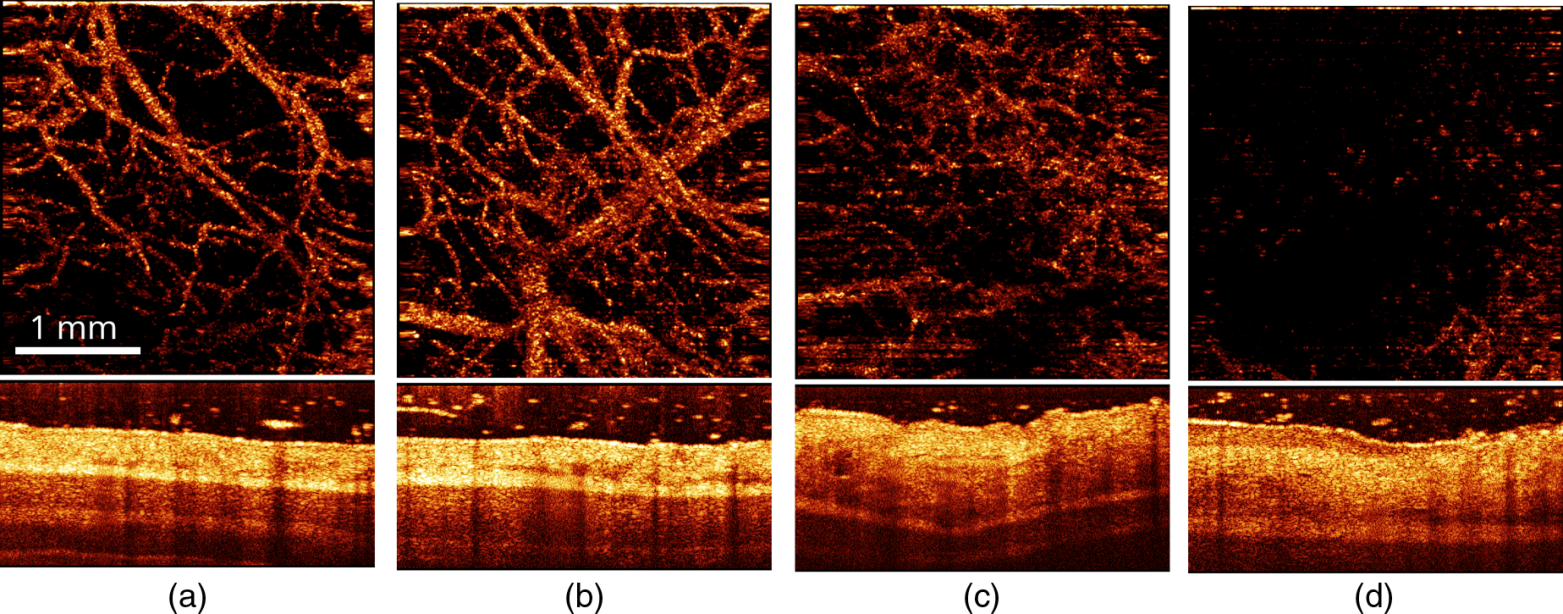
(Top) Angiographic *en-face* projection and (bottom) structural OCT images of rabbit ear (thick compartment) obtained from different animals in (a) 4 and (b) 7 days after IRR_b100 exposure (irradiation-only, $100\;{J/\mathrm{cm}}^{2}$, 405 nm) and from the same animal in (c) 4 and (d) 7 days after IRR_b150 exposure (irradiation-only, $150\;{J/\mathrm{cm}}^{2}$, 405 nm). The size of top and bottom images is 3×3 and 3×1  mm, respectively.

The irradiation-only blue light regimes IRR_b100 and IRR_b150 demonstrated preservation of microcirculation up to 7 days after exposure [Figs. 11(a) and 11(b)], except one animal after IRR_b150 exposure, which demonstrated stasis in 7 days [Fig. 11(d)], although in 4 days for this site, the microcirculation was preserved [Fig. 11(c)]. Structural OCT images for this animal revealed morphological alterations in 4 days after irradiation [Fig. 11(c)] that led to severe changes [Fig. 11(d)] with formation of a rigid scab.

On the contrary to the blue light regimes, the PDT_r100 and PDT_r150 regimes as well as IRR_r100 and IRR_r150 exposures did not induce any significant alterations in rabbit ear tissue, and the reaction was limited by edema of moderate degree, and no cases of microcirculation disturbance were registered.

**Fig. 12:**
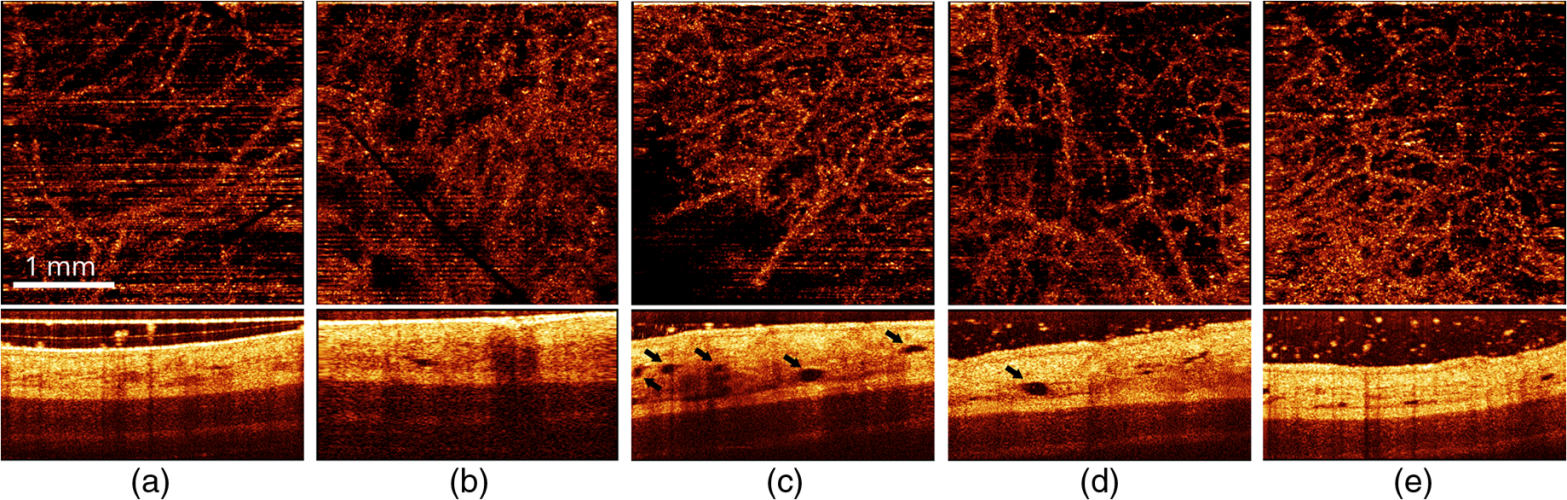
(Top) Angiographic *en-face* projection and (bottom) structural OCT images of rabbit ear (thin compartment) (a) prior to, (b) immediately after, and in (c) 1, (d) 4, and (e) 7 days after IRR_rb150 exposure (irradiation-only, $75+75\;{J/\mathrm{cm}}^{2}$, 660+405  nm). The areas corresponding to a pronounced activation of lymphatic system are marked with black arrows. The size of top and bottom images is 3×3 and 3×1  mm, respectively.

Similar to the case of the equal doses delivered with red light, no morphological changes were observed in all animals with PDT_rb100 and PDT_rb150 regimes (except one site for PDT_rb150 regime, where severe changes were detected), as well as with IRR_rb100 and IRR_rb150 exposures. It is worth mentioning that, in one animal with IRR_rb150 exposure, a pronounced activation of lymphatic system was observed (Fig. 12).

**Fig. 13:**
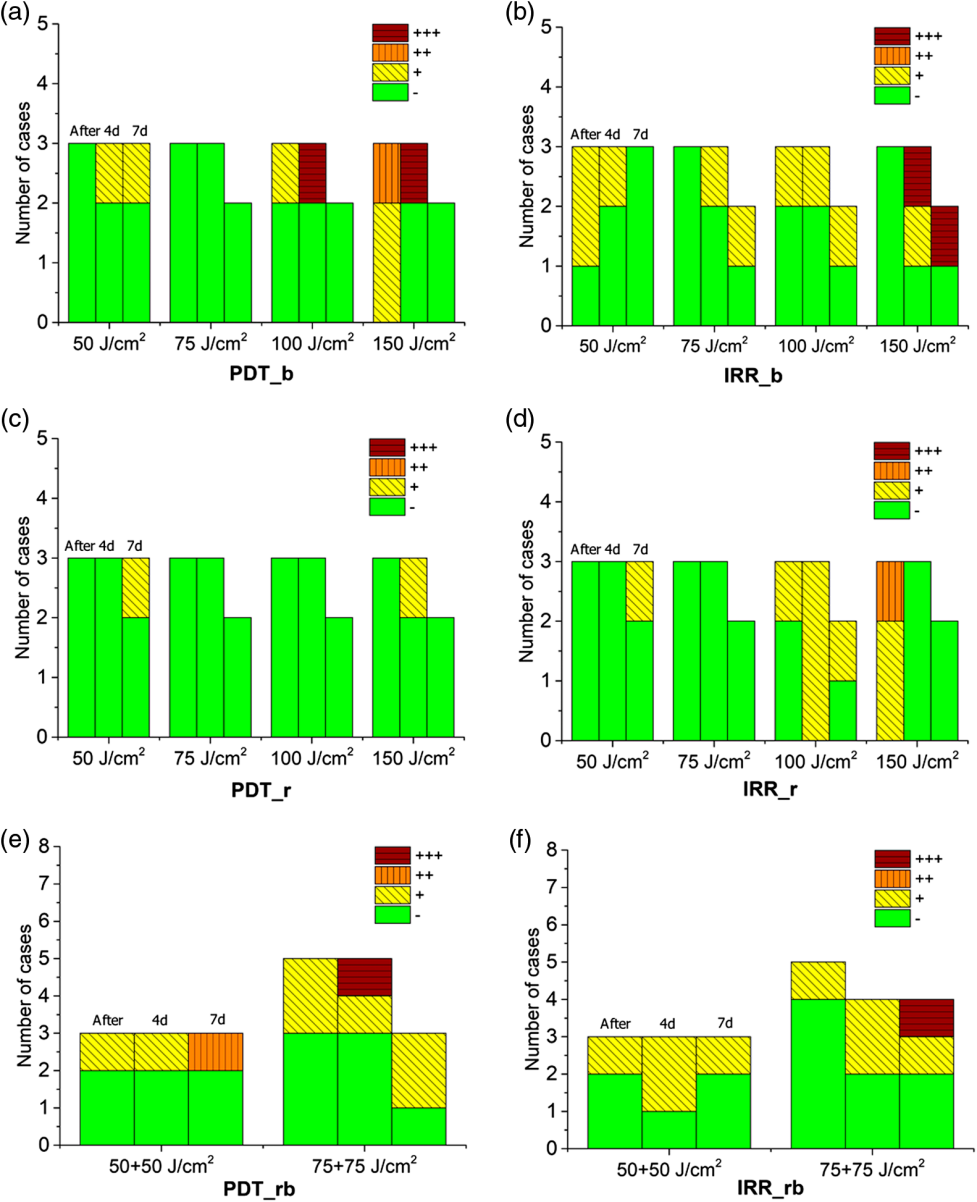
Comparative analysis of the PDT procedure outcomes revealed by OCT images. PDT regimes with (a) blue light (PDT_b), (c) red light (PDT_r), and (e) the two wavelengths (PDT_rb). Irradiation-only exposures with (b) blue light (IRR_b), (d) red light (IRR_r), and (f) the two wavelengths (IRR_rb). Each group of bars describes the outcome for the indicated light dose immediately after, in 4 and 7 days after procedure. Color encodes the edema degree and the observed structural changes: green (−), no edema or weak edema manifestation; yellow (+), moderate edema; orange (++), pronounced edema; dark-red (+++), severe changes such as disappearance of layered structure and/or stasis.

Normal tissue reactions to both PDT and IRR exposures are summarized in Fig. 13. To quantify the observed changes, primarily edema, in the OCT images, we introduced the semiquantitative approach using a four-level classification of the procedure outcome: (−) no effect or minor edema, (+) moderate edema, (++) pronounced edema, and (+++) severe changes. Generally, PDT procedures with red light result in weaker tissue response as compared to blue light and the combination of red and blue lights. PDT procedures also provide less tissue reaction in 7 days after exposure as compared to the same light doses delivered without PS administration. This effect may be explained by the fact that, in the case of PDT, a part of irradiation is absorbed by PS, thus inducing photodynamic reaction, while without PS, the entire absorbed light dose contributes to direct impact to the tissue.

### 3.6 Histological Study of the Photodynamic Therapy Procedure Outcome

To trace the reversible and irreversible changes in the normal tissue of a rabbit ear, biopsy samples were taken from intact tissue as well as from exposed tissue in 1, 4, and 7 days after PDT and IRR exposures and analyzed using H&E staining.

**Fig. 14:**
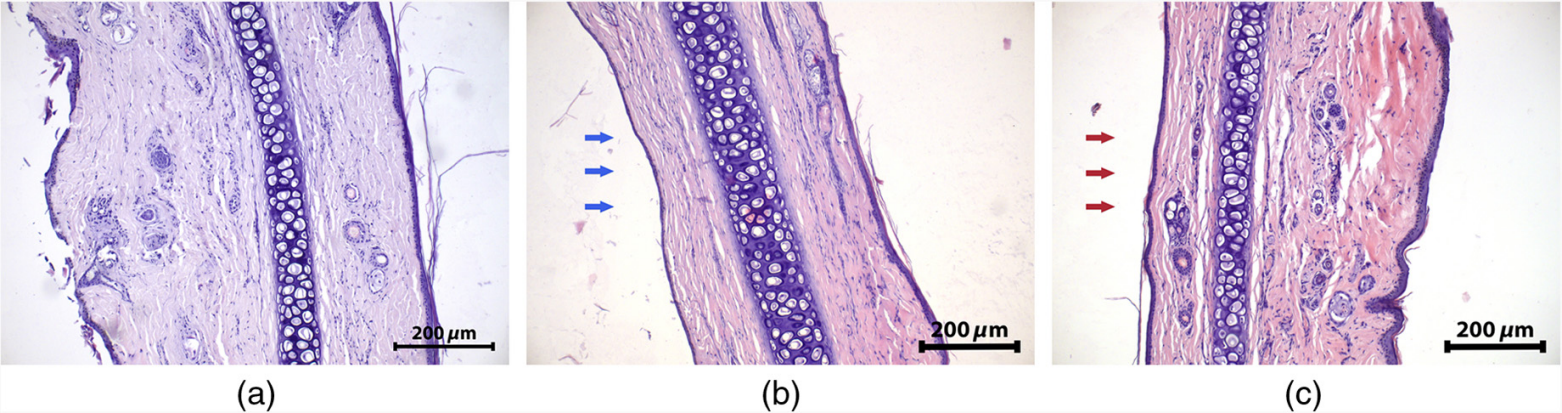
Histological H&E images of a rabbit ear tissue: (a) intact tissue, 1 day after (b) PDT_b50 ($50\;{J/\mathrm{cm}}^{2}$) and (c) PDT_r50 ($50\;{J/\mathrm{cm}}^{2}$, 660 nm). Arrows in (b) and (c) indicate the site of irradiation.

Typical H&E images of a rabbit ear intact tissue and 1 day after PDT_b50 and PDT_r50 procedures are shown in Fig. 14. For PDT_b50 procedure, histological examination revealed edema manifestation primarily in the superficial layer from the irradiated side, while PDT_r50 procedure led to a more uniform edema observed over the entire depth. It is explained by the character of blue versus red radiation attenuation with the depth predicted by the preliminary Monte Carlo simulations.

**Fig. 15:**
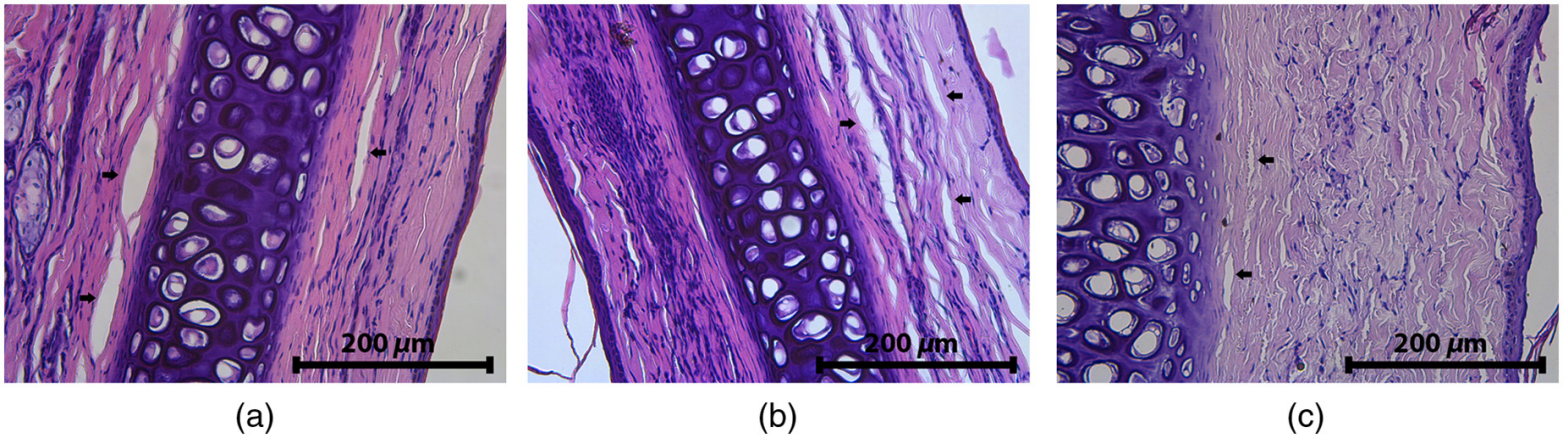
Histological H&E images of a rabbit ear tissue (a) 1, (b) 4, and (c) 7 days after PDT_b50 procedure ($50\;{J/\mathrm{cm}}^{2}$, 405 nm). Arrows represent areas of edema.

Figure 15 demonstrates the dynamics of changes in case of PDT_b50 procedure. Samples are presented at high magnification in comparison with previous ones that allows for more detailed investigation. The edema relaxation was observed in 7 days after PDT procedure that is in good agreement with the results of OCT monitoring. Similar effects were observed for other monowavelength regimes. Pronounced edema manifestation was observed in histological images for all the considered regimes in 4 days after exposure. In 7 days, a decrease in edema manifestation was observed for all the considered exposure regimes, except PDT_b100 and PDT_b150 regimes, for which focal necrosis (not shown) was revealed, which can be associated with the alteration observed with OCT.

**Fig. 16:**
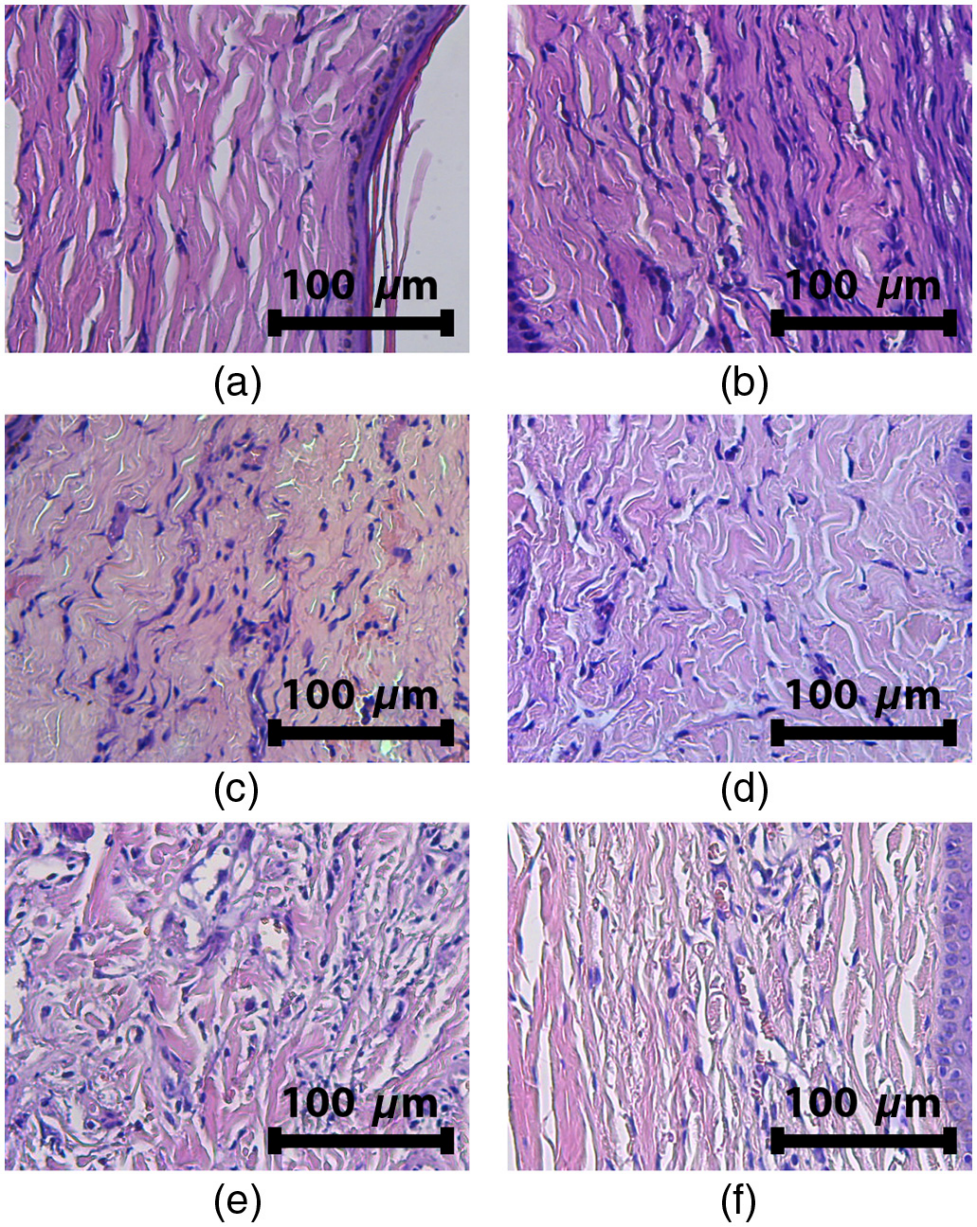
H&E images of a rabbit ear tissue in 4 days [(a) and (b)] and 7 days [(c) and (d)] after PDT_rb150 ($75+75\;{J/\mathrm{cm}}^{2}$, 660+405  nm) procedure [(a) and (c)] and IRR_rb150 (irradiation-only, $75+75\;{J/\mathrm{cm}}^{2}$, 660+405  nm) exposure [(b) and (d)]. H&E images in 7 days after (e) IRR_b150 and (f) IRR_r150 exposures.

Figures 16(a) and 16(c) demonstrates H&E images after PDT_rb150 procedure acquired in 4 and 7 days, where the degree of manifestation of edema decreases by the seventh day. Similar observation was obtained for IRR_rb150 [Figs. 16(b) and 16(d)], however, with more pronounced residual degree of edema. Even higher manifestation of edema is observed for IRR_b150 [Fig. 16(e)] and IRR_r 150 [Fig. 16(f)] exposures delivered at single-wavelength regimes.

Obviously, histological inspection is a more sensitive tool for edema detection and evaluation as compared to OCT, since edema is observed in the cases where OCT does not demonstrate edema signs. This is explained by the typical scale of edema manifestations, which is of the order of units of microns, as can be evaluated from the histology images (Fig. 15). This scale is below the spatial resolution of the employed OCT setup, whereas only large edema areas with the size of tens of microns can be visualized by OCT. Nevertheless, due to the advantages of noninvasive real-time monitoring, the ability of OCT to detect strong edema manifestations is of high importance for evaluation of tissue response to the PDT procedure.

## 4 Conclusions

The studied PDT and irradiation-only regimes included doses of 50, 75, 100, and $150\;{J/\mathrm{cm}}^{2}$ in single-wavelength mode and doses of 100 and $150\;{J/\mathrm{cm}}^{2}$ in dual-wavelength mode (equal contribution of blue and red lights).

Dual-wavelength FI was employed for monitoring the PS distribution in treated tissue. In the course of all considered PDT regimes, effective PS photobleaching (>40%) was revealed. Based on the red-to-blue fluorescence signal ratio dynamics, dual-wavelength FI allowed the estimation of the depth of PDT action.

Noninvasive IR temperature monitoring demonstrated that, although all the considered regimes caused statistically significant temperature increase, the absolute temperature value did not exceed 40°C. A comparative analysis of temperature increase as a result of different exposure regimes with equal doses was performed.

OCT monitoring performed up to 7 days after the exposure demonstrated that the reaction of normal tissue to the procedure was manifested by edema, activation of lymphatic system, and higher exposure doses were accompanied by stronger manifestations. OCT angiography demonstrated that all the considered regimes did not affected tissue microcirculation, except for the dose of $150\;J/{\mathrm{cm}}^{2}$ delivered with blue light, which induced stasis. Generally, relaxation of the tissue reaction was observed in 7 days after the PDT procedure. PDT procedures with red light induced weaker tissue response in comparison to PDT regimes with blue light and the combination of red and blue lights. The comparison of the PDT procedures and the exposures without PS administration demonstrated weaker tissue reaction to PDT in 7 days after exposure.

Histological examination revealed that, due to the limited penetration of blue light into the tissue, the irradiation at λ=405  nm leads to edema primarily from the irradiated side, while application of red light (λ=660  nm) leads to uniform edema distribution within tissue, which is in agreement with preliminary Monte Carlo simulations. For all considered regimes, edema was observed in 4 days after exposure, followed by relaxation of manifestations by the seventh day. According to the histological analysis, the manifestation of edema as a result of light exposure was stronger in the irradiation-only group, as compared to the PDT group. These observations are in agreement with the results of OCT monitoring.

The performed analysis allows the prediction of the reaction of normal tissues to PDT procedures with chlorin-based PSs performed both with red and blue lights, which is important for antibacterial PDT and antiaging PDT, as well as for predicting the reaction of normal tissues neighboring the tumor to the antitumor PDT.
